# Nutritional assessments and interventions in head and neck, esophageal, rectal and lung cancers undergoing anticancer treatments: a literature review

**DOI:** 10.3389/fonc.2025.1676305

**Published:** 2025-11-06

**Authors:** Valentina Casalone, Chiara Lazzari, Elena Fassi, Vanesa Gregorc

**Affiliations:** 1 Clinical Nutrition and Dietetics Unit, Candiolo Cancer Institute, FPO-IRCCS, Candiolo, Italy; 2 Department of Medical Oncology, Candiolo Cancer Institute - FPO - IRCCS, Candiolo, Italy; 3 Medical Oncology Unit, Department of Medical and Surgical Specialties, Radiological Sciences, and Public Health, University of Brescia, ASST Spedali Civili, Brescia, Italy

**Keywords:** malnutrition, nutritional intervention, immunonutrition, anticancer treatments, head and neck cancer, lung cancer, rectal cancer, esophageal cancer

## Abstract

In the last decades, greater toxicities deriving from anticancer treatments (especially chemo and/or radiotherapy), along with cancer sites, increase the risk of nutritional status impairment. This condition should be avoided because it could determine early therapies’ interruptions and worse clinical outcomes. In this review, we aim to provide an overview of the current evidence obtained from Pubmed and Embase databases assessing the role of nutritional assessments and interventions during anticancer treatments, with a particular focus on immunonutrition. Actual evidence suggests that nutritional practices are different worldwide, however, it is essential to define an adequate and standardized nutritional evaluation including at least food intake estimation, anthropometric measurements and body composition analysis. Nutritional interventions should always include intensive counseling and, in some cases, the prescription of specific dietary supplements. Nowadays, immunonutrition formulas represent a promising tool to improve many nutritional and treatment outcomes, but further studies are still necessary to define an evidence based clinical practice.

## 1 Introduction

Chemotherapy and radiotherapy, especially combined, have become, over the recent years, established standards of care for many solid cancers due to their remarkable benefits in terms of survival and local disease control (1). However, the potentiation of cytotoxic effects inevitably induces greater toxicities: as a consequence, patients could experience a worsening of the nutritional status, which finally leads to malnutrition (2, 3).

In the present review we have analyzed solid cancers for which the effects of an immunonutritional intervention during oncology therapies have been most frequently studied. Head and neck squamous cell cancers (HNSCC) are frequently associated with malnutrition (as reported in about 60% of patients) because of the close proximity to vital organs normally involved in eating functions (4). In locally advanced forms, CRT represents the gold standard option in adjuvant and curative settings and it is associated with a better progression-free survival (PFS) and a better overall survival (OS) versus radiotherapy (RT) alone (5, 6). The tradeoff for improved disease control with CRT are the increased acute and late toxicities such as mucositis, dysphagia, odynophagia and xerostomia: all these symptoms further reduce the ability to achieve an adequate oral food intake (7, 8). Patients who report malnutrition or unintentional weight loss (even in case of baseline overweight or obesity) usually present poorer treatment outcomes, higher morbidity and mortality rates and lower Quality of Life (QoL) (7–9).

Locally advanced non–small cell lung cancers (LANSCLC) are even associated with higher malnutrition rates (almost 70% of patients as reported in some studies), which are mainly due to the presence of fatigue, pain, loss of appetite and coughing (10, 11). Surgery is an option only for a selected subset of patients, whereas curative CRT still represents the standard of care: it has been reported to be more efficacious than RT alone, especially in stage III (12, 13). However, this treatment commonly causes toxicities such as dry mouth, early satiety, dysphagia, nausea, vomiting and dysgeusia which are all recognized as nutrition impact symptoms (NIS) (14).

Due to the direct involvement in food behavior, the invasive nature of the disease and the presence of dysphagia, also esophageal cancer is associated with high malnutrition rates, accounting for 67-85% of patients (15). When surgery is not possible, CRT represents the main approach for its efficacy in improving clinical outcomes (16). However, nutritional status during therapies is frequently worsened due to the presence of esophagitis, nausea and dysgeusia (17).

On the other hand, malnutrition rates of locally advanced rectal cancer (LARC) patients are often underestimated (18). CRT also represents the actual standard of care for LARC, before or after radical surgery, because it significantly improves local disease control and recurrence rates (19). However, acute and late toxicities reported by patients (i.e. diarrhea, loss of appetite, cystitis, bowel dysfunction, fecal incontinence) could increase the risk of malnutrition which could itself strongly affect treatment tolerability and postoperative complications (18, 20, 21).

The present narrative review aims to address the following topics:

- role and methods of nutritional assessment and interventions during chemotherapy (CT) and/or RT treatment in patients with cancers presenting high risk of malnutrition;
- future directions of nutritional interventions in this setting.

A bibliographical search was conducted in PubMed and Embase using a Boolean search string combining keywords related to “gastrointestinal cancer” OR “lung cancer” OR “head and neck cancer” AND “chemotherapy” OR “radiotherapy” OR “chemoradiotherapy” AND “malnutrition” OR “body composition” OR “muscle mass” OR “sarcopenia” OR “nutritional screening” OR “dietary intake” OR “handgrip” OR “nutritional counseling” OR “immunonutrition”.

We selected studies in English language published between 2000 and 2025. The inclusion criteria included both longitudinal or cross-sectional observational studies; we also reported controlled or non-controlled trial regarding different nutritional interventions. Systematic reviews and meta analysis were excluded by the results, but were considered in the discussion.

No quantitative pooling of data was performed due to substantial heterogeneity across the included studies in terms of design, population, interventions and outcomes. Instead, a structured qualitative synthesis was conducted to identify, organize, and critically interpret the main findings emerging from the literature.

## 2 Nutritional assessment during antineoplastic treatments

Both cancer and antineoplastic treatments are frequently responsible for determining malnutrition, which is associated with treatment dose reductions, higher toxicities, QoL worsening and lower survival. It is estimated that about 20% of patients die from the effects of this condition (22–24). However, despite the recognized and documented importance of this problem, cancer related malnutrition still remains underestimated and undertreated worldwide (15, 22, 25, 26). This phenomenon may be attributed to: the heterogeneity of the actual literature, about the definition of malnutrition and the implementation of different nutritional practices (27).

The easiest and fastest method to assess nutritional status is through the implementation of specific validated screening (28). The most used ones are: the Nutritional Risk Screening (NRS-2002), which evaluates body mass index (BMI), weight loss and food intake (29); the Patients-Generated Subjective Global Assessment (PG-SGA) which considers weight loss, NIS, food intake and physical activity (30); the MUST score whose parameters are BMI, unintentional weight loss in the previous 3–6 months and the potential acute effect of illness on food intake (31); and the Mini Nutritional Assessment (MNA), that examine weight loss, BMI and the ability in performing normal daily activities (32). Clinical nutrition societies developed the GLIM (Global Leadership Initiative on Malnutrition) criteria which allow a diagnosis and gradation of malnutrition after a nutritional screening has reported a risk. The diagnosis requires at least one phenotypical criteria (non-volitional weight loss, low BMI, reduced muscle mass) and one etiological criteria (reduced food intake or assimilation, systemic inflammation) (33). However, in the usual clinical practice, nutritional status is often evaluated only through body weight (BW) and BMI assessment (34). The European Society for Clinical Nutrition and Metabolism (ESPEN) suggested that these measures could be biased due to the impossibility to evaluate body composition (BC) (22). In fact, even if the muscle mass, usually defined as lean body mass (LBM), is often decreased because of disease and/or therapies and/or inadequate nutrition, BW and BMI are not representative of its variations (35, 36). The identification of muscle wasting could be even more difficult in case of obesity (reported in 40-60% of cancer patients (4): literature show that BW stability could mask clinically significant skeletal muscle depletion, leading to the condition known as sarcopenic obesity (37–39). According to the EWGSOP (European Working Group on Sarcopenia in Older People), sarcopenia is defined as “a progressive and generalized skeletal muscle disorder that is associated with increased likelihood of adverse outcomes including falls, fractures, physical disability and mortality” (40). A rapid diagnostic test to evaluate the risk of sarcopenia is the SARC-F questionnaire which investigates five items (strength, assistance in walking, rise from a chair, climb stairs and falls) (41). Further options to better explore BC in patients presenting decreased muscle quality and quantity should be carried out performing computerized tomography, magnetic resonance scans or bioelectric impedance analysis (BIA) (4). The latter is widely used, because of its simplicity and non-invasivity in evaluating the electrical characteristics of the human body in the clinical routine (42, 43). Phase angle (PA) is the most important recognized parameter derived from the BIA test and it is considered a prognostic factor of survival and an indicator of cellular health (44). Another data of clinical relevance in treatment outcome prediction is the assessment of skeletal muscle function (SMF), whose surrogate markers are handgrip strength (HGS) and handgrip endurance (HGE) (45). According to EWGSOP, the muscle strength cut-offs are 27 kg for men and 16 kg for women (40). Loss of skeletal muscle mass (SMM) and reduced muscle strength, which are both shown in many cancer patients, are considered negative prognostic factors since they are strongly associated with fatigue, disability, treatment-related complications, low QoL and survival rates (especially in elderly patients) (46, 47).

### 2.1 Results

The majority of observational studies reporting the prevalence of malnutrition, before and after antineoplastic treatment and according to validated screening, are shown in **Table 1**. The most used tools are the NRS-2002 and the PG-SGA. Although HNSCC patients usually report high malnutrition prevalence since the baseline, a study conducted in 55 patients reported that about 70% of patients were not malnourished or at risk of developing this condition; however, after treatment the percentage decreased at less than 20%, showing the great impact of antineoplastic treatment on the nutritional status of these patients (48). Similar baseline data were shown for LARC patients, but the impact of RT was lower than those in HNSCC. However, their results suggested that malnutrition was still present at 6–8 weeks after the end of treatment and it was correlated with post-operative complications (anastomotic leakage) (18). On the contrary, esophageal cancer and NSCLC reported high malnutrition rates even at baseline and nutritional status was furtherly deteriorated with antineoplastic treatments (14, 17). Unfortunately, the absence of similar studies do not allow to compare results in the same settings.

**Table 1:** Characteristics of the studies assessing prevalence of malnutrition.

Author, year, country (ref)	Study design	Study population	Treatment	Number of patients	Methods to detect malnutrition	Results
Stegel, 2016, Slovenia (48)	Prospective, longitudinal, observational study	Men > 18 years, with non metastatic HNSCC	RT alone or in combination with CT	55	NRS-2002 [Cachexia = weight loss > 5% in 6 months; BMI < 20 kg/m2 and SMM depletion with sarcopenia and weight loss > 2%.]	Pre-treatment: - 69.1% well nourished, 16.4% malnourished, 14.5% cachectic. Post-treatment: - 16.4% well nourished, 45.4% malnourished, 38.2% cachectic.
Yamano, 2016, Japan (18)	Prospective, longitudinal, observational study	Patients with stage III-IV LARC	CRT	49	NRS-2002	Pre treatment: - 77.6% well nourished, 22.4% malnourished. Post-treatment: - 40.8% well nourished, 59.2% malnourished.
Lin, 2020, China (14)	Prospective, longitudinal, observational study	Patients > 18 years, with advanced NSCLC	CT	465	PG-SGA	Pre-treatment: - 23% well nourished, 65.6% moderately malnourished, 11.4%severely malnourished. Post-treatment: - 13.3% well nourished, 52.9% moderately malnourished, 33.8% severely malnourished.
Movahed, 2020, Iran (17)	Cross-sectional survey study	Patients > 18 years, with esophageal cancer	RT alone or in combination with CT	71	PG-SGA	Before treatment: - PG-SGA score > 8 (requiring nutritional interventions): 84.5% -PG-SGA score 4-8 (requiring dietary modification): 12.8%

Food intake is one of the essential elements for the maintenance of a good nutritional status, however few studies have explored its dynamic change during treatments (**Table 2**). Despite the different settings, both Zhuang and Movahed reported that patients’ intake were under the recommended ESPEN threshold even before starting treatments and oral intake progressively decreased during all the course of therapies (17, 22, 49). The study of Guren showed that also LARC patients experience a reduction of oral food intake during treatment (50), but they did not report data concerning kilocalories or proteins/kg of weight: for this reason, it is not possible to correlate these results with the previous studies.

**Table 2:** Characteristics of the studies assessing food intake.

Author, year, country [ref]	Study design	Study population	Treatment	Number of patients	Methods to detect food intake	Results
Zhuang, 2023, China (49)	Prospective, longitudinal, observational study	Patients > 18 years, with HNSCC	RT	462	24-hour dietary recall	Pre-treatment (T1): 23.07 kcal/kg/day and 0.86 g proteins/kg/day Mid-treatment (T2): 17.9 kcal/kg/day and 0.75 g proteins/kg/day Post-treatment (T3): 15.65 kcal/kg/day (p<0.001) and 0.65 g proteins/kg/day (p<0.001)
Movahed, 2020, Iran (17)	Cross-sectional survey study	Patients > 18 years, with esophageal cancer	RT alone or in combination with CT	71	24-hour dietary recall	Pre-treatment: -19.5 kcal/kg/day and 0.56 g proteins/kg/day; - insufficient calorie and protein intake: 55.7% and 72.7%. Post-treatment:: - 18.3 kcal/kg/day and 0.66 g proteins/kg/day; - insufficient calorie and protein intake: 58.7% and 89.1%. Insufficient intake of vitamin D, vitamin E, zinc and calcium both before and after treatment
Guren, 2006, Norway (50)		Patients > 18 years, with LARC	RT	31	7-day food intake registration	Mean reduction of 4 kcal/kg from baseline to the end of treatment; decrease of 15, 10 and 8% respectively for fats, proteins and carbohydrates. Dietary intakes of fiber, calcium, iron, vitamin D and a-tocopherol under the recommended values.

Body changes during anticancer treatments have recently started to be explored in studies analyzing BC (**Table 3**). Several studies have shown that many parameters such as SMM, LBM, fat free mass (FFM) and fat mass (FM), change at different time points during treatments and, for example in HNSCC patients, these variations could not be effectively monitored only using BW and BMI (43, 50). According to Zhuang et al., the majority of weight loss is represented at the beginning by SMM and, in a second time, by FM (49). However, both parameters are significantly reduced during treatment, both in HNSCC and esophageal cancers (17, 48, 51, 52). The loss of muscle mass has also been correlated with a deterioration of muscle function (reported as HGS) (54). The statistically significant reduction of this parameter during treatments was correlated with many clinical outcomes (53, 54). The study of Yamanaka also found a positive correlation between HGS and PA: they showed that low PA is associated with worse outcomes (55).

**Table 3:** Characteristics of the studies assessing anthropometry, body composition and muscle function.

Author, year, country [ref]	Study design	Study population	Treatment	Number of patients	Tools to detect BC/muscle function	Results
Zhuang, 2020, China (49)	Prospective, longitudinal, observational study	Patients > 18 years, with HNSCC	RT	462	Lookin’ Body 120 analyzer (Inbody) - BIA technology	Changes from baseline to the end of treatment: -4.6 kg BW, -1.9 kg FM, -2.6 kg FFM, -1.5 kg SMM; 70% of patients reported > 5% weight loss.
Cao, 2021, China (51)	Prospective, observational study	Patients > 18 years, with HNSCC	RT alone or in combination with CT	287	BIA - Inbody Co., LTD, 120 Seoul, Korea	SMM: - pre-treatment: 26.4 kg; - post treatment: 24.6 kg (p<0.001). Diagnosis of sarcopenia (AWGS Criteria (56)): - pre-treatment: 24.4%; - post-treatment: 46.7% (p<0.001).
Chauhan, 2020, India (52)	Short-term, longitudinal cohort study	Patients > 30 years, with HNSCC	CRT	19	OMRON Karada Scan Hbf 375 Body Composition Monitor Jamar handgrip dynamometer	SMM: - pre-treatment= 29.9 kg; - 3rd week= 28.7 kg; - 7th week= 26.4 kg (p<0.05). HGS: - pre-treatment= 26.1 kg; - 3rd week= 23.3 kg; - 7th week= 20.4 kg (p<0.05). Diagnosis of sarcopenia (SARC-F score (57)): -pre-treatment: 31.5%; -post-treatment: 89.4%
Movahed, 2020, Iran (17)	Cross-sectional survey study	Patients > 18 years, with esophageal cancer	RT alone or in combination with CT	71	BIA Tanita BC-418 Jamar handgrip dynamometer	BW -pre-treatment= 57.02 kg; -post-treatment= 54.6 kg (<0.001). FM -pre-treatment= 24.11%; -post-treatment= 22.8% (<0.001). HGS -pre-treatment= 43.2 kg; -post-treatment= 36.1 kg (<0.001).
Huang, 2021, China (54)	Single-center prospective, observational study	Patients with stage II-IV HNSCC	CRT	82	64-Slice CT (SOMATOM Sensation 64, Siemens, Munich, Germany) Jamar^®^ Plus + Digital Hand Dynamometer (JA Preston Corporation, Jackson, MS, USA)	HGS reduction was correlated with grade 3–4 leukopenia (OR = 0.92, p=0.007) and any grade 3–4 toxicity (OR = 0.94, p=0.013).
Burtin, 2020, the Netherlands (53)	Prospective cohort study	Patients > 18 years, with NSCLC	RT alone or in combination with CT	936	BIA Omron Healthcare Group Jamar hydraulic hand dynamometer	Low FFMI (27%): -correlation with female sex (p<0.029), worse performance status (p<0.0002), lower BMI (p=0.015), handgrip weakness (p<0.0001) and OS (21 *vs* 27 months; p=0.049). Handgrip weakness (26%): -correlation with male sex (p<0.001), worse performance status (p<0.001), age (p<0.0001), lower BMI (p=0.015), low FFMI (p<0.0001), OS (19 *vs* 27 months; p <0.0001). Combined presence of hand grip weakness and low fat free mass index (FFMI) was a strong prognostic factor for OS in patients with good performance status (HR = 1.79, p < 0.001).
Stegel, 2016, Slovenia (48)	Prospective, longitudinal, observational study	Men > 18 years, with non metastatic HNSCC	RT alone or in combination with CT	55	BodyStat QuadScan 4000 (Douglas, Great Britain) Baseline digital hydraulic dynamometer (NexGenErgonomics Inc., Montreal, QC, Canada)	PA: -difference between well-nourished and malnourished/cachectic patients (5.49° *vs* 4.99°, P = 0.045); -no differences between pre/post-treatment. HGS: -pre-treatment: 42.3 kg; -post-treatment: 40.8 kg (p=0.04).
Yamanaka, 2022, Japan (55)	Prospective, observational study	Patients with HNSCC	CRT	96	DSM-BIA using InBodyS10 (InBody, Tokyo, Japan) Dynamometer (Takei Scientific Instruments, Niigata, Japan)	PA: -positive correlation with HGS (R = 0.649), SMMI (R = 0.62). Low-PA group reported higher incidence of anemia (52 *vs* 17%), aspiration (17 *vs* 1%) RT interruption (17 *vs* 3%), low 3-years OS (47 *vs* 81%, p<0.05).

## 3 Nutritional interventions during antineoplastic treatments

International guidelines suggest that interventions during CT and/or RT should always include nutritional counseling (NC) in order to achieve an adequate food intake and to prevent weight loss and treatment interruptions (22). It is intended as a two-way interaction where a patient and a nutritionist interpret nutritional assessment and identify problems, goals and future steps. In most cases, nutritional counseling is accompanied by the prescription of oral nutritional supplements (ONS) (58, 59).

Nutritional status and BC (intended as fat and muscle stores) are able to influence the functioning of the innate and the adaptive immune system through the modulation of hormones and cytokines production (60). Macro or micronutrients deficiency can cause an impairment of cell-mediated immunity, phagocyte function, complement system, secretory IgA antibodies concentration and cytokine production: as a consequence, the immune system loses the elements used to start an effective response (61). One of the mediators implicated in the reduction of immune cell number is leptin, a hormone secreted by adipose tissue, whose levels are decreased in malnourished patients (62). Leptin acts directly on CD4+ T cells which are responsible for inflammatory cytokine secretion: in presence of malnutrition, studies have reported a reduced inflammatory cytokines production of IFN-γ and IL-2 (62). On the other hand, abundant abdominal fat of obese patients is associated with higher levels of inflammatory cytokines, adhesion molecules and prothrombotic molecules that leads to a condition of chronic low grade inflammation (61). In fact, adipose tissue-resident immune cells are mainly characterized by activated macrophages which leads to an increase in TNF-α and other inflammatory molecules such as IL-1β, IL-6 and IL-12 (60). TNF-α, which is a well-known pro-inflammatory cytokine essential for the acute phase reaction, can be produced by adipose tissue and is decreased in case of weight loss (63, 64). IL-6 is a cytokine responsible for promoting T cell survival, resistance to apoptosis and CD4+ T cell differentiation that leads to the production of the proinflammatory cytokines IL-17 and IFN-γ (65). In parallel, also cancer-induced systemic inflammation promotes proinflammatory cytokines (IL-6, TNF-α, IFN-γ) production that activate the hypothalamic-pituitary adrenal axis: the consequent relapse of catabolic stress hormones (cortisol, adrenaline and glucagon) cause muscle wasting through the interruption of the proteostasis (66).

The altered immune response of cancer patients due to both disease and nutritional status, has led to the exploration of specific nutrients with immunomodulating properties known as immunonutrients (67). The rationale of use is to obtain a beneficial effect on the immune system, especially in controlling systemic inflammation (68). Immunomodulatory components that can be used in cancer patients are arginine, glutamine, omega 3 fatty acids, nucleotide acids and pre-and probiotics (69). Arginine is a basic amino acid that participates in the synthesis of cellular proteins, nitric oxide (NO), glutamic acids, proline, glutamine and creatine (70). In physiological conditions is normally sufficient, whereas in clinical conditions with increased catabolism such as cancer, the body’s demand for this amino acid is higher (70–72). Its immuno-nutritional properties are due to the ability in influencing thymic production of T cells, granulocytic phagocytosis, functioning of NK cells and anti-tumor cytotoxicity (70–72). Glutamine is a conditionally essential amino acid that participates in the ATP biosynthesis and in the protection of the body against reactive oxygen species. Its immunomodulating role is due to its ability in inhibiting the proinflammatory cytokines production (73). However, due to the abundance in the human body and its water insolubility property, it is not normally used in the commercial immunonutrition formula (69, 73). Omega-3 fatty acids, that normally refers to eicosapentaenoic acid (EPA) and docosahexaenoic acid (DHA), are able to inhibit the activity of NF-kB, a nuclear transcription factor responsible of the activation of proinflammatory cytokines production (such as IL-6, IL-1, IL-10, TNF-a) and C-reactive protein (PCR) (74–76). Nucleotide acids of RNA and DNA are normally derived from human body synthesis and from diet, but conditions of catabolism could increase their demand. They affect maturation, activation and proliferation of lymphocytes and support the regeneration of intestinal villi. Normally, nucleotides are found in specific immunonutrition formulas that also contain arginine and omega-3 fatty acids (69). Pro-and prebiotics are also considered immunonutrients respectively for their capacity of decrease proinflammatory cytokines (IL-8, TNF-α) and to modify intestinal microbiome that is responsible for the maintenance of the intestinal barrier (69, 77, 78).

### 3.1 Results

In the last decades, many studies have started to explore the role of different nutritional interventions during anticancer treatments. **Table 4** summarizes the efficacy of NC, ONS and immunonutrition during CT and/or RT. Intensive NC was correlated with better nutritional and treatment outcomes, such as lower therapy interruptions or complications (79, 80). Isenring et al. and Cardellini et al. added the prescription of ONS, reporting significant benefits in many nutritional status parameters and even in better OS (81, 82). Due to the direct effect of omega-3 fatty acids in improving inflammation, some studies have explored their use in patients undergoing CT or RT treatments: both Faber and Roca-Rodrìguez reported an improvement of inflammatory parameters (83, 84). Furthermore, they were associated with higher protein-calorie intake, greater weight gain and lower toxicities (85). In recent years, the promising results in cancer surgery of a commercial formula enriched with omega-3, nucleotides and arginine, has gradually lead to an increasing number of studies analyzing its effects in the CRT setting (86–90). Machon investigated its efficacy on inflammation, oxidative stress and toxicities in HNSCC patients, reporting an initially different modulation of the inflammatory state; however, the absence of a control group was a big limitation of this study (86). Boisselier and colleagues replicated the study using a control group: they showed a significant improvement of OS and PFS although compliance was not optimal (87). Other studies also showed a great impact on nutritional status and immune cells modulation (88–90).

**Table 4:** Characteristics of the studies assessing the efficacy of different nutritional interventions.

Author, year, country [ref]	Study design, (phase)	Study population, oncological treatment	Number of patients	Nutritional intervention (start-end)	Dose of specific nutrients	Adherence (%)	Significant primary/secondary outcomes	Sponsors
van den Berg, 2010, the Netherlands (79)	Prospective clinical study	Patients > 18 years, with II-IV stage HNSCC; RT alone or in combination with CT	38 (19 *vs* 19)	Intensive NC *vs* standard care (before the start of RT - 2 months after the end)	–	–	Lower prevalence of malnutrition (intended as unintended weight loss ≥5% in one month or ≥10% in six months) 2 weeks after treatment (0% *vs* 28%, p=0.02) and lower weight loss 2 months after treatment (p=0.03).	No
Paccagnella, 2010, Italy (80)	Retrospective study	Patients with HNSCC; CRT	66 (33 *vs* 33)	Early nutritional intervention *vs* no nutrition program (before the start of CRT – after the end of treatment)	–	–	Lower weight loss (-4.6 kg *vs* -8.1 kg, p<0.01), RT breaks > 5 days (30.3% *vs* 63.6%, p< 0.01), RT delay for toxicities (4.4 *vs* 7.6, p<0.05), unplanned hospitalizations (16.1% *vs* 41.4%, p=0.03).	No
Isenring, 2004, Australia (81)	Prospective, randomized, controlled trial	Patients > 18 years, without enteral or parenteral support; RT	60 (29 *vs*31)	Intensive weekly NC with ONS in case of need *vs* standard care (within the first 4 days of RT - 12 weeks from the start)	–	–	Lower weight loss (-0.4 kg *vs* -4.7 kg, p<0.001), nutritional status deterioration (-1.6 *vs* +3.1 PG-SGA score, p=0.02) and QoL decrease (p=0.009).	Yes
Cardellini, 2024, Italy (82)	Observational, prospective study	Patients > 18 years, with HNSCC cancers; CRT	60	NC with energy-dense and/or high protein ONS or artificial nutrition in case of need (start of CRT - 3 months after the end)	300–400 kcal and 20–40 g of proteins per bottle (based on individual requirements)	–	Maintenance or improvement of anthropometric parameters; weight at the end predicted disease free (p=0.007) and OS (p=0.015)	Yes
Sànchez-Lara, 2014, Mexico (85)	Randomized, controlled trial	Patients 18–80 years, with stage III-IV NSCLC, ECOG 0-2; CT	92 (46 *vs* 46)	EPA-enriched ONS *vs* isocaloric diet (7 days before the start of CT - second CT cycle)	590 Kcal, 30 g of proteins, 2 g of EPA (2 bottles per day)	>73%	Higher energy (p<0.001) and protein (p<0.001) intake; greater LBM gain (+1.6 kg *vs* -2 kg, p=0.01); reductions of fatigue, loss of appetite and neuropathy (p<0.05).	Yes
Faber, 2013, The Netherlands (83)	Prospective, randomized, controlled trial	Patients > 18 years, with solid cancers; RT	38 (19 *vs* 19)	Medical food enriched with fish oil *vs* an isocaloric product (first day of RT - seven days after the beginning)	650 Kcal, 40 g of proteins, 2,4 g of EPA, 1,2 g of DHA (400 ml per day)	92%	Higher incorporation of EPA (p<0.001) and DHA (p<0.05) in white blood cells; reduction of serum levels of the inflammatory mediator PGE2 (p<0.01).	Yes
Roca-Rodrìguez, 2013, Spain (84)	Prospective, randomized, controlled study	Patients 18–80 years, with stage III-IV ear, nose and throat cancer; RT alone or in combination with CT	26 (13 *vs* 13)	Enteral formula enriched with n-3 polyunsaturated fatty acids and antioxidant *vs* standard enteral formula (14 days after RT start - 60 days after treatment)	288 kcal, 16 g of proteins, 1.1 g of EPA/240 ml (based on individual requirements)	–	Lower decrease of BMI, hematocrit, erythrocyte, eosinophil and albumin levels and lower increase of creatinine and urea levels; earlier normalization of metabolic, inflammatory and oxidative stress parameters at the end of treatment.	No
Machon, 2012, France (86)	Prospective non- controlled pilot study (phase 2)	Patients 18–70 years, with stage III or IV HNSCC; CRT	31	Commercial immunonutritional formula (5 days before each cycle of CT)	909 Kcal, 50 g of proteins, 3 g of EPA+DHA, 11.4 g of arginine, 7 g of glutamine, 1.3 g of RNA (3 sachets per day)	–	Reduction of CRP (p=0.002) and α-1 acid glycoprotein (p=0.002) levels after 5 days of oral supplementation; no significant variation of inflammatory markers or mucositis incidence.	Yes
Boisselier, 2020, France (87)	Double-blind multicenter study (phase 3)	Patients 18–75 years, with HNSCC; CRT	180 (90 *vs* 90)	Commercial immunonutritional formula *vs* an isocaloric, isonitrogenous formula (5 days before each cycle of CT)	909 Kcal, 50 g of proteins, 3 g of EPA+DHA, 11.4 g of arginine, 7 g of glutamine, 1.3 g of RNA (3 sachets per day)	65% (> 75% compliance)	No differences in incidence of grade 3–4 mucositis; improvement of 3 years-OS in compliant patients (81 *vs* 61%, p=0.034) and of PFS (73 *vs* 50%; p=0.012). Compliance ≥75% immunonutrition group: 66% of patients.	Yes
Chao, 2020, Taiwan (88)	Retrospective study	Patients 18–80 years, BMI 16-28, receiving enteral feeding > 7 days, with HNSCC or esophageal cancer; CRT	88 (44 *vs* 44)	Enteral immunonutritional formula *vs* an isocaloric, isonitrogenous polymeric formula (more than seven days during CRT)	1740 ± 293 Kcal, 77 ± 15 g of proteins, 11 g of arginine, 1.4 g of ribonucleotides, 1.8 g of EPA, 1.2 g of DHA	–	Improvement of NRI in malnourished patients (97.3 *vs* 98, p=0.021); significant differences in BW (+0.9 kg *vs* -0.6 kg, p< 0.05), BMI (+0.4 *vs* -0.4; p<0.05) and total proteins (+0.13 g/dL *vs* -0.39 g/dL, p<0.05).	No
Vasson, 2014, France (89)	Multicenter, randomized, double blinded, controlled trial	Patients > 18 years, with HNSCC or esophageal cancer and WHO Performance status ≤ 2; CRT	37 (18 *vs* 19)	Enteral immunonutritional formula *vs* an isocaloric, isonitrogenous formula (at least from 5 days before the beginning of CRT - end of treatment)	Minimum 1515 Kcal, minimum 56 g of proteins, 13 g of arginine, 1.3 g of nucleotides, 3.4 g of EPA+DHA (minimum 1500 ml per day)	–	Significant improvement of BW (p < 0.05), BMI (p < 0.05) and LBM (p < 0.05) and plasma antioxidant capacity (p < 0.05); increase of albumin and NRI in malnourished patients; maintenance of functional capacity (p < 0.05).	Yes
Talvas, 2015, France (90)	Multicentre, randomized, double blinded, controlled (phase 2 trial)	Patients > 18 years, with HNSCC or esophageal cancer and WHO Performance status ≤ 2; CRT	18 (13 *vs* 15)	Enteral immunonutritional formula *vs* an isocaloric, isonitrogenous formula (at least from 5 days before the beginning of CRT – end of treatment)	Minimum 1515 Kcal, minimum 56 g of proteins, 13 g of arginine, 1.3 g of nucleotides, 3.4 g of EPA+DHA (minimum 1500 ml per day)	–	Stability of CD4+/CD8+ T-lymphocyte counts ratio and of the production of pro-inflammatory prostaglandin E2; increased response of immune cells.	Yes
Gül, 2022, Turkey (91)	Prospective, single-center, controlled study	Patients > 18 years, with LARC; after neoadjuvant CRT	40 (20 *vs* 20)	Immunonutrition group *vs* standard nutritional supplement (7 days before surgery – day of surgery)	Not reported	–	Lower CD4/CD8 ratio (p=0.026)	No

## 4 Discussion

Cancer can frequently be associated with nutritional status impairment, especially in case of direct or indirect involvement with food behavior (4). For this reason, the majority of the studies have focused their attention in HNSCC, esophageal and LANSCLC, where over 10% of patients are already severely malnourished even before starting active antineoplastic treatments (14, 17, 48). Literature has also widely reported that therapies could induce a further compromission of patients’ nutritional status. The overcited studies have shown that malnutrition rates gradually increase even between the middle and the end of therapies, underlining the necessity to repeatedly evaluate patients’ nutritional status during treatments (14, 48). An adequate nutritional intake is essential for the maintenance of a good nutritional status: however, food intakes were often much lower than the 25–30 kcal/kg/day and the 1-1.5 g proteins/kg/day recommended by the ESPEN guidelines regarding nutritional needs on cancer patients (as cited in the second chapter) (22) These results were particularly relevant in HNSCC patients at the end of treatment (49, 51).

In the past, BW was used as the main parameters for nutritional status assessment; however, in the last few years, different studies have started to carry out BC analysis because of the interesting discovery about the correlation of these parameters with many clinical outcomes (49, 53–55). For example, Zhuang reported a statistically significant decrease in SMM during treatment: according to the ESPEN Guidelines, this parameter is the main element of cancer-associated malnutrition (22, 49). Even LBM and body cellular mass (BCM), which are frequently reduced during CRT and that corresponds to poor muscle mass and wasting of lean tissue, are associated with treatment toxicities and higher risk of recurrence (43). Another important bioelectrical parameter to be considered is PA, whose value is related with higher risk of sarcopenia and low survival. Yamanaka and colleagues were the first to show a correlation between PA and toxicities or treatment interruption in HNSCC patients during CRT. However, larger studies are required to confirm these results (55) New studies are also correlating handgrip parameters with BC. During combined CRT, HNSCC, esophageal cancer and NSCLC patients usually report significant reductions of HGS: this parameter is related with LBM, PA and BCM, denoting an association between muscle function and BC (17, 45, 48, 52–54). Significant association between HGS and treatment toxicities and OS were also reported, underlining the prognostic role of this parameter (52–54). It is important to consider that some studies are not completely comparable due to the use of different cut-off values. Further reviews are needed to homogenize cut-offs even between different populations. Furthermore, it must be underlined that, at the moment, the majority of these results derived from studies exploring CT or RT alone, whereas combined treatments were less explored. Given the increased toxicity rates of this therapeutic approach, it must be considered the higher risk of further nutritional status impairment, in terms of weight loss, oral food intake derangement or BC worsening. For this reason, personalized NC during CRT should always be carried out in order to ensure nutritional requirements (even through the prescription of oral, enteral or parenteral support, in case of need) and to improve nutritional status, QoL, toxicity management, PFS and OS (22, 24, 92). The overall positive effects of nutritional interventions during CRT were mainly attributed to the use of omega-3-enriched ONS, suggesting the benefit of targeting metabolic alterations in parallel with energy and protein supplementation (82, 83, 93). The ESPEN guidelines suggest the use of these fatty acids in advanced cancer patients undergoing CT and at risk of malnutrition to improve appetite, food intake, LBM and body weight; whereas immunonutrition is at the moment recommended only for upper gastrointestinal cancer patients undergoing surgical intervention (22). However, thanks to its efficacy in improving BC, inflammation and nutritional status, recent studies are exploring new settings of use, especially during CRT for HNSCC, where malnutrition and NIS rates are very high. All the studies reported in literature used a commercial immunonutrition formula. Despite the different use of this product (in terms of type and length of administration), every study reported significant improvement in many outcomes, such as the reduction of inflammation parameters or the increase in BW, albumin, OS and antioxidant capacities (86, 87, 89–91). However, the compliance to this commercial formula currently constitutes the greatest limitation for is use: the percentage of adherent patients was about 60-70%, with lower values in already malnourished patients (85, 87). Furthermore, according to different systematic reviews, the presence of sponsorship from pharmaceutical companies increases the risk of study bias (86, 87, 89, 90). Other limits were represented by the absence of nutritional counseling and the administration of these products only in the 5 days before treatment, which could not be sufficient to improve nutritional outcomes, as underlined by Caccialanza and colleagues (94). Despite the heterogeneity of the studies, the systematic review of Zhang et al. stated that immunonutrition administered in CRT could reduce the incidence of grade ≥3 oral mucositis, grade ≥3 diarrhea, grade ≥3 esophagitis and the risk of losing >5% of body weight, opening to a new era of nutritional supplementation during antineoplastic treatments (95).

## 5 Conclusions

In summary, literature suggests that nutritional status impairment is strongly associated with worse treatment outcomes, as frequently reported by HNSCC, esophageal, LANSCLC and LARC patients undergoing CRT. Severe toxicities deriving from this combined therapeutic approach could further induce malnutrition with consequent risk of early therapies interruption. Observational studies all agree in showing a derangement of nutritional status, but the heterogeneity of methods used for its assessment, do not consent to provide specific recommendations to define the optimal clinical practice. Wider studies are necessary for better understanding the interconnections between nutritional status, inflammation, immunity and treatment outcomes in order to define more tailored nutritional interventions. At the moment, nutritional counseling and nutritional supplements remain efficient and affordable practices to improve or maintain an adequate nutritional status. Immunonutrition represents a promising tool but, at the moment, few data are available in the setting of chemoradiotherapy: further studies are needed to strengthen current results, find more-compliant approaches and define an evidence-based practice for this nutritional tool.
